# Designing knowledge-driven digitalization: novel recommendations for digitally supported multi-professional collaboration

**DOI:** 10.3389/fdgth.2025.1511973

**Published:** 2025-04-07

**Authors:** Oliver Meindl, Sarah Peuten, Xena Striebel, Henner Gimpel, Christoph Ostgathe, Werner Schneider, Tobias Steigleder

**Affiliations:** ^1^ FIM Research Center for Information Management, Augsburg, Germany; ^2^ Branch Business & Information Systems Engineering, Fraunhofer Institute for Applied Information Technology FIT, Augsburg, Germany; ^3^ University of Augsburg, Augsburg, Germany; ^4^ Chair of Sociology, University of Augsburg, Augsburg, Germany; ^5^ Chair of Digital Management, University of Hohenheim, Stuttgart, Germany; ^6^ Palliativmedizinische Abteilung, Comprehensive Cancer Center CCC Erlangen-EMN, Universitätsklinikum Erlangen, Friedrich-Alexander-Universität Erlangen-Nürnberg, Erlangen, Germany

**Keywords:** multi-professional collaboration, knowledge-driven digitalization, co-creation, recommendation, collaboration support system, palliative care, action design research

## Abstract

**Introduction:**

Palliative care is based on the principle of multi-professional collaboration, which integrates diverse competencies and perspectives to provide holistic care and support for patients and their relatives. In palliative care teams, there is an intensive exchange of information and knowledge; however, current documentation and hospital information systems often fall short of meeting the specific demands for effective collaboration and dynamic communication in this field.

**Methods:**

This action design research study is based on the three-and-a-half-year interdisciplinary research project PALLADiUM and aims to demonstrate the added value of knowledge-driven digitalization.

**Results and discussion:**

Our study provides novel recommendations for digitally supported multi-professional collaboration tailored to the specific requirements of palliative care and similar fields. Based on the analytical distinction between ‘information’ and ‘knowledge,’ we present design recommendations for co-creative, knowledge-driven development processes and multi-professional collaboration support systems. We further illustrate how these recommendations have been implemented into a functional technical demonstrator and outline how our results could impact future digitalization initiatives in healthcare.

## 1 Introduction

Palliative care (PC) is characterized by a holistic, person-centered approach that addresses the subjective needs of patients and their relatives. Instead of focusing on cure or prevention, PC aims to maintain or increase quality of life and to ease physical, psychological, social, and spiritual suffering. To address these various perspectives adequately, PC is based on the principle of multi-professional collaboration, integrating various competencies and viewpoints to provide comprehensive care for patients and their relatives (1–3). Due to the specific contextual factors and professional ethos in this field of work, PC has traditionally been somewhat distant from technology and digitalization and has been transformed in this regard to a much lesser extent compared to other medical fields (4). Supporting systems introduced in the course of digitalization initiatives have to adequately consider the specific conditions of the respective work environment and be useful to the extent that work performance and collaboration are perceivably improved. Additionally, they have to be easy enough to use in order to be accepted by the various involved healthcare professionals (HCPs) (5, 6). Due to the enormous daily workload in healthcare, the expected effort for these systems also plays a vital role (5, 7, 8). Given the highly multi-professional nature of PC, the limited available time resources, and the increased skepticism of PC practitioners towards technology use, it makes digitalization initiatives particularly challenging, especially as all end-users need to be co-creatively involved (4, 9, 10). Despite these drawbacks, the number and penetration of supporting digital health systems are constantly increasing. Examples include mobile health technologies for symptom management or monitoring vital and movement parameters, telemedicine, and electronic documentation systems (11, 12). These applications primarily address the level of interaction between patients and HCPs or inter-sectoral collaboration (13, 14) rather than internal team collaboration itself. On PC units in Germany, digital support of clinical routines has only just begun, primarily driven by hospital-wide initiatives to enhance efficiency and quality standards. Yet, current systems often fall short of meeting the specific demands emerging through collaborative work practices and the heterogeneous ways of communication in PC, which is an overarching challenge for healthcare involving multiple stakeholders (15). The relatively low level of digitalization and the lack of suitable collaboration-oriented digital systems present an outstanding opportunity to investigate how collaboration support systems and digitalization processes can be sensibly designed at an early stage.

It is insufficient to purely design technical systems to successfully integrate and incorporate technology into PC practice. Instead, digitalization initiatives should be considered as an interplay of technological aspects, educational elements (e.g., the enablement to use the systems) along with relevant information and knowledge aspects (e.g., documentation and communication about patients). This approach aligns with other digital transformative approaches in healthcare (16). In a PC setting, there is an intensive exchange of ‘information’ and ‘knowledge’ between the various professionals comprising the multi-professional team, as well as between these professionals and other key stakeholders, including the patient and their family (4). An essential guiding research assumption in the interdisciplinary research project ‘Palliative Care as a Digital Working World’ (PALLADiUM) is the analytical distinction between ‘information’ and ‘knowledge’ (17, 18). Information is objective, respectively, actor-independent, and available to anyone with access to it. It is primarily decontextualized and stands for itself, although it requires different contextual knowledge to be understood. Knowledge is the completion of information by subjects or actors, depending on the respective context or situation, through individually and/or collectively shared meanings and interpretation frameworks. Knowledge is, therefore, just as culture-dependent as it is actor-bound and subjective. There is communicatively conveyed or communicable knowledge (e.g., experiential knowledge) and implicit knowledge that is difficult or impossible to make explicit (e.g., intuition) (19). For a smooth and effective collaboration, it is crucial to have pertinent information and the knowledge of team members made available and utilized to achieve a shared understanding of the respective patient situation (20).

An integrated set of recommendations is needed to improve the adequate consideration of ‘knowledge’ (in the analytical distinction between knowledge and information) that is crucial to PC work practices but often neglected within digitalization initiatives, especially in the hardly digitally supported area of multi-professional collaboration. Hence, we state the following research question:

Which recommendations can be derived from knowledge-driven digitalization for multi-professional collaboration in PC?

To answer this question, we pursue a rigorously field-specific and empirically based design process, understanding the participation of practitioners as process- *and* outcome-oriented. This means that we follow a co-creational, continuous participation approach (21–24). Co-creation is an approach that is known to foster patient-centeredness, mutual exchange of knowledge (e.g., working practices) between various stakeholders, and trust, thereby enabling future practitioners to make better use of the system to be designed (21, 25, 26). The focus is not only on the outcome (i.e., the system) but also on the process itself (27). In doing so, PALLADiUM serves as a case study focusing on multi-professional collaboration in inpatient PC. It demonstrates that palliative-specific digitalization initiatives, which cater to team members’ diverse needs and daily work dynamics, can enhance communication and collaboration processes, improve information and knowledge flow, and improve patient care (19, 20). While previous digitalization initiatives have focused primarily on information management and information exchange, the project demonstrates that it is essential to consider relevant knowledge aspects.

The following sections present design recommendations (DRs) for co-creational, knowledge-driven development processes within multi-professional collaboration. It further offers concrete DRs for multi-professional collaboration support systems. We additionally discuss transfer possibilities beyond PC to other healthcare contexts where multi-professional collaboration is essential.

## 2 Method

This study is based on the three-and-a-half-year interdisciplinary research project PALLADiUM, for which a published study protocol gives insight into its objectives and participating disciplines (i.e., social scientists, information systems engineers, and PC experts) (28). The research project aimed to support multi-professional communication and collaboration in PC utilizing digital technologies. We set out to iteratively develop and evaluate a collaboration support system based on practitioners’ perspectives and relevancies. PALLADiUM investigates the daily practical collaboration within the team, explicitly focusing on the HCPs as a multi-professional team in the PC unit and their collaboration in everyday care. Correspondingly, patients, their families, and the public were not involved. However, a second practice perspective was obtained from another PC unit. In such practical endeavors, action design research has been demonstrated to effectively explore new digital technologies or complex socio-technical phenomena (29). A positive ethics vote (IRB Friedrich-Alexander-Universität Erlangen-Nürnberg: #168_21 B) allowed us to collaboratively co-create a demonstrator for the collaboration support system between practitioners and researchers within the PC ward of the University Hospital of Erlangen in Germany. Following Sein et al. (29), our research process had four stages: I–IV.

(I) Problem formulation. Over two years, off-site dialogues and on-site ethnographic fieldwork with employees of the University Hospital of Erlangen served to identify deficiencies in information and knowledge exchange, as well as gaps in information transfer within the multi-professional collaboration on the PC ward. From these findings, multi-professional collaboration challenges were subsequently derived, and digital potentials were explored. Monthly meetings within the research team helped to deduce the initial requirements for a multi-professional collaboration support system. The scoping of the literature supported our intermediate findings for the problem from a theoretical point of view.

(II) Building, intervention, and evaluation. To develop a multi-professional collaboration support system, we co-created and evaluated a collaboration support system demonstrator at the PC ward in Erlangen. Therefore, we applied three steps. Step 1) We started with ethnographic fieldwork (30–32), observing team interactions over four 14-day cycles. This allowed us to access implicit knowledge and understand routine collaboration practices. All team members (*n* = 33) participated in the study after giving informed consent. During the research stays, the researchers were granted access to all internal team meetings, participated in rounds, and were allowed to move freely within the ward (except for patient rooms). They spent time in the team members’ offices and were present at therapeutic interventions by individual arrangement. All team members were systematically considered in a conceptual sense, as all team members, when present, were potentially observed. However, it is essential to note that certain professional groups were more strongly represented (nurses, physicians) in terms of numbers than others, and some professional groups do not work on the ward every day. This inevitably led to varying observation durations and opportunities for involvement. Nevertheless, formative evaluation throughout the entire project period, in which continuous feedback and contributions were possible, was ensured through the extensive participant observations on-site. Two researchers recorded detailed field notes on-site, later elaborated into field protocols (30). The qualitative data collection and analysis were conducted according to Grounded Theory, characterized by an iterative approach alternating between data collection and analysis. Grounded Theory does not refer to a strict method but rather to a research perspective that follows certain methodological principles within a qualitative research design and can be adapted and specified in its approach and methodological implementation to address practical problems within the respective field of practice. Key elements include continuous conceptualization and the increasing ‘condensation’ and abstraction of raw data (field protocols and focus group transcripts) through open, axial, and selective coding, constant comparison, theoretical sampling, and the use of memos. Grounded Theory thus aims to generate empirically grounded analytical knowledge (rather than knowledge derived from abstract theories) and develop domain-specific theories that can be used to address practical problems within the field of practice. Despite the explicitly inductive approach, prior knowledge (e.g., literature, professional and personal experiences) and knowledge gained in the field throughout the current study are seen as central resources, reflected in guiding research assumptions and the use of ‘sensitizing concepts’ (33–36). Coding was carried out continuously, with data from earlier fieldwork being recoded in light of new insights. The coding process was systematic, but it did not follow a quantitative logic (such as standardized, verifiable intercoder reliability) since no existing theory was applied or tested; rather, the aim was to engage in empirically grounded, domain-specific ‘theory-building’. The collected data material was independently coded by at least two researchers and a total of four researchers, with results regularly discussed within the interdisciplinary research team and in interpretive sessions with other social scientists. The focus was on intersubjective comprehensibility and a transparent, research-driven approach.

This iterative approach identified key workflows, communication and collaboration tools, competencies, information and knowledge needs. Focus groups were held to discuss and refine identified challenges of multi-professional collaboration, involving team members from all professional groups (physicians, nurses, spiritual caregivers, psychologists, case managers/social workers, physical and music therapists). Step 2) Based on these insights, we developed requirements for the collaboration support system, which involved discussions with ward executives and team members to pinpoint potential enhancements. Focus groups were organized to discuss these requirements and gather feedback on how they could improve workflows. All focus groups were scheduled at times when participation was possible for all professional groups, and we ensured that each professional group participated in at least one focus group. However, due to daily dynamics, planned participations sometimes had to be canceled at short notice. By incorporating an additional practical perspective at a second location (the PC unit of the University Hospital Augsburg), results and requirements for the investigation field at the University Hospital Erlangen were also reflected upon and assessed for generalizability. Utilizing the requirements and transferring them to features, we developed a technical demonstrator suitable for smartphones and desktop computers. Step 3) Anonymized case presentations were employed in the final summative evaluation, highlighting specific challenges in team collaboration. In a four-day on-site test, three case vignettes—patient trajectory vignettes based on the empirical data from the field cycles and enriched with written documentation from the hospital information system (HIS)—were distributed to nine team members from six professions during their shifts following an evaluation protocol. Participation was once again open to all professional groups, and those who were present and had the time and interest were welcome to participate. The team tested the demonstrator on smartphones and desktop computers (depending on preference) using fictitious patients, simulating a realistic environment. A focus group also evaluated the demonstrator through hands-on sessions and feedback discussions.

(III) Reflection and learning. This phase parallels the former two. Our approach was marked by the continuous integration of feedback from members of the PC ward team, which facilitated continuous discussions and reflections within the interdisciplinary research team. The resulting findings guided and shaped our research process by ingraining the reality of clinical practice.

(IV) Formalization of learning. Formalizing the insights and learnings gathered during our research process, we could induce and abstract two sets of recommendations. First, we could derive recommendations for co-creational, knowledge-driven development processes in multi-professional collaboration and transfer them to digitalization contexts in healthcare. Second, we induced DRs for collaboration support systems for multi-professional environments comparable to PC.

## 3 Results

### 3.1 Design recommendations for co-creational, knowledge-driven development processes in multi-professional collaboration

The iterative and ethnographically orientated research design emphasizes our commitment to an open, co-creational, and continuous participatory approach (21–24). The multi-professional team and its members, each bringing their background, expertise, and perspectives, were central to the entire research process (37). They are key knowledge actors who not only serve as knowledge holders and transmitters but are also actively involved as knowledge producers. Team members provided both structured (through focus groups) and unstructured, spontaneous feedback (during on-site visits), along with concrete implementation ideas and recommendations for adjustments and changes. Participation was not limited to specific time points or explicit topics but was ongoing and encompassed all aspects deemed significant by the participants. Through extensive observations and conversations with the members of the multi-professional team on the PC unit, we identified and validated challenges in daily collaboration, understood organizational and team-related interrelationships, and explored opportunities for a precisely tailored, digitally assisted collaboration support system. The overview in Table 1 highlights the primary *challenges*, shows that specific challenges emerge as *undesired effects* of these fundamental issues, outlines potential *ideas and solutions* to address them, and presents the overarching *purposes*. Both technical and process-related ideas and solutions are therefore considered.

The interplay of the elements, including challenges, undesired effects, solution ideas, and purposes, as exemplified in the context of multi-professional collaboration in the PC unit, demonstrates that researchers and team members are engaged in a reciprocal and comprehensive learning process. This process cannot be confined to individual research steps or work packages. Instead, it must be integrative and continuous to address the inherent complexities of multi-professional collaboration and to understand results in their respective contexts and impacts.

Based on Table 1 and its preceding explanations, Table 2 provides generalized DRs for co-creative, knowledge-driven development processes in the context of multiprofessional collaboration.

**Figure 1:**
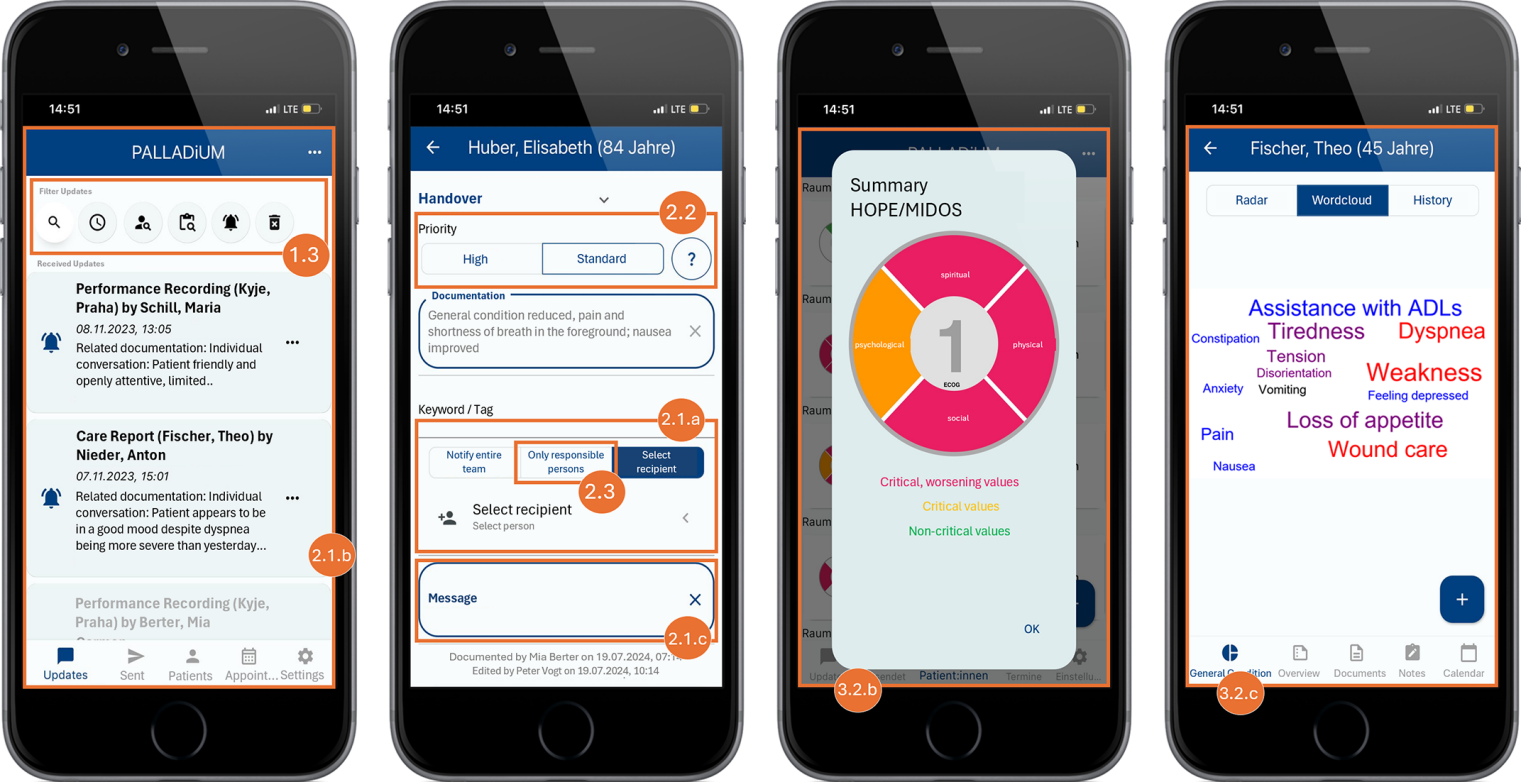
Translated screenshots of the mobile app. Note: The patient data presented is fictitious.

The recommendations outlined are also reflected in the specific DRs provided in Section 3.2, as well as in the examples of implemented features (e.g., Table 3, DRs on *Information and Knowledge Exchange*, and Figure 1, Number 2.1.c). Tables 1, 2 thus represent the empirical foundation for the technical ‘translation’ and concretization of these recommendations.

**Table 3:** Design recommendations for multi-professional collaboration support systems.

DR	Area	Mechanism	Aim
1.1	Access and usability	Enable seamless access and functionality that adapts to diverse devices and operating systems.	To lower barriers for all professions to document.
1.2		Facilitate contributions from all professional roles through a unified and consistent user interface across the whole system.	To democratize the system and promote equal and easy access to information and knowledge.
1.3		Implement a user-friendly search and filter functionality on shared team knowledge and existing documentation about patients.	To ensure efficient information retrieval and minimize time spent seeking documented information or knowledge.
2.1	Information and knowledge exchange	Enable information sharing and knowledge exchange in asynchronous dialogues.	To foster decentralized collaboration, continuous reduction of information and knowledge gaps, and a shared and holistic understanding of patient cases.
2.2		Ensure real-time delivery of relevant information and knowledge to team members.	To promote effective care coordination, enabling prompt responses to patient needs.
2.3		Provide transparent and easily accessible information regarding the responsibility of professionals and the availability of resources.	To facilitate well-coordinated and informed care based on patients’ needs considering the available resources.
3.1	Information and knowledge tangibility	Provide visual representations that enable a detailed view of historical patient data.	To facilitate well-informed decision-making leveraging the vast number of historical insights on patients.
3.2		Process and aggregate information and knowledge in a clear and verifiable manner.	To support efficient analysis of patient's current status and effective decisions on treatment strategies.
4.1	Data integration	Provide continuous access to up-to-date, centralized patient data through seamless integration with existing information systems.	To create a comprehensive information base that facilitates efficient data management between systems.
4.2		Enable users to record and store personal notes anywhere and anytime digitally.	To enhance individual knowledge retention based on captured observations and reflections.
5.1	Documentation flexibility	Allow for tailoring information input fields to match the stationary conditions and team-specific requirements.	To facilitate accurate and structured documentation considering specific organizational needs.
5.2		Allow for the flexible updating of data fields beyond standard medical data to encompass crucial general information.	To ensure a comprehensive and more holistic view of the patient, which will lead to more personalized care.
5.3		Provide multiple ways to document information and knowledge.	To allow to document in individual ways, thus reducing ambiguity and potential for miscommunication.

**Table 1:** Challenges in multi-professional palliative care collaboration, undesired effects, solution ideas, and purposes.

Challenges	Undesired effects	Ideas/solutions	Purposes
Different perspectives and knowledge divergences • Treatment priorities • Medication • Recency bias • Communication opportunities • Continuing care options • Role of relatives	Team-related effects • Different case/situation definition • Different treatment approaches • Team dissent/team irritation Potential patient-related effects • Risk of disruption in (home) care • Risk of patient and caregiver distress	New messaging features • Supplementary information/context • Explanations/comments • Impressions • Pending items, unresolved issues Modified symptom presentation • Symptom development • Counterintuitive representations Enhanced search/filter and clustering features; tags New, expanded and more palliative-specific documentation fields • Patient overview: Preferences, dislikes, infections, allergies, responsibilities, family situation • Patient deceased (+ room information) • Individualized and profession-specific views • Updates, changes Reflection • What should be consistently known by the entire team/specific professional groups/specific team members? • (Unchallenged) practices, norms • Meeting formats and times	Making profession-specific relevancies and knowledge divergences within the team visible, reflexively accessible and thus workable; Promoting a shared understanding of what should/should not be done, when, why and by whom Enhancing transparency regarding procedures, decisions, outstanding tasks and treatments Bridging asynchronicity and reducing information and knowledge gaps Taking into account typical dynamics and rapid/situational changes Strengthening the multi-professional approach and promoting interprofessional discussion Facilitation of new structured spaces for exchange/deliberation Objectification Egalitarianism Strengthening certainty of interpretation and certainty of action
Oral communication vs. written documentation Deficiencies of the existing HIS	Information/knowledge gaps • Hospice registration completed? • Speaking valve requested? • Planned/possible continuation of care • Can the patient eat by himself/herself? Delays Double documentation and extra work		
Team aspects • Avoiding team friction/team stabilization, confirmation practices • Positions/roles in the team; hierarchies • Informal practices/norms	Pending decisions Unclear responsibilities Interpretation and action uncertainty Delays Information/knowledge gaps		

**Table 2:** Design recommendations for co-creational, knowledge-driven development processes in the context of multi-professional collaboration.

Mechanism	Aim
Ensure an open and flexible development process.	To establish a foundation for co-creation and to accommodate evolving insights and requirements.
Consistently adapt to the conditions, formal workflows, and informal work practices on-site.	To ensure that all ward members have equal participation opportunities and access to the development process (e.g., no inequities due to differing work hours/presence). To build and foster trust and acceptance.
Create continuous opportunities for interaction and exchange between the development team and ward members.	
Systematically consider relevant knowledge aspects for the multi-professional collaboration and incorporate them into the development process (e.g., experiential knowledge, existing case knowledge, current impressions).	To make profession-specific relevancies and knowledge divergences within the team visible, reflexively accessible, and thus addressable within the development process. To promote a shared understanding of what should or should not be done, when, why, and by whom. To increase the certainty of interpretation and certainty of action.
Be aware of profession-specific and situation-related information and knowledge needs.	
Consider hierarchies and power dynamics, such as differing spatial and speaking arrangements and positions or roles within the team.	To strengthen the multi-professional approach, enhance transparency, and contribute to egalitarianism in the development process and ultimately in multi-professional collaboration.
Take seriously and reflect on the limits of what can and should be sensibly digitalized.	To acknowledge the importance and function of face-to-face interaction in multi-professional collaboration.

### 3.2 Design recommendations for multi-professional collaboration support systems

We designed a multi-professional collaboration support system following the above-described co-creative, knowledge-driven digitalization process. To implement and demonstrate these design concepts, we developed a technical demonstrator^1^. The development was designed as a ‘greenfield’ approach, deliberately avoiding constraints of existing infrastructure and tailoring the system for the PC context. Given this approach, time-intensive technical aspects like data architecture, security concepts, or application programming interface integrations did not have to be designed, leading to a stronger focus on digitally improving collaboration. Yet, the system was conceptualized to interface with existing HIS while addressing critical challenges in multi-professional collaboration. The primary goal was to enhance interpretive and operational certainty through three key design objectives: entering relevant information and knowledge, obtaining relevant information and knowledge, and analyzing & aggregating available information and knowledge. During the design, particular emphasis was placed on the situational-appropriate visualization of the patient's historical and current symptom burden to support the joint decision-making of the PC team. Key requirement areas identified included IT-supported human-to-human information exchange, documentation, user interaction, and knowledge foundation. Additional established digital collaboration features, such as a shared calendar, further enhanced the initial requirement list for the development. To ensure accessibility across different platforms, the demonstrator was developed using Flutter^2^ with the programming language Dart^3^, enabling an operating system agnostic deployment (e.g., on an Android smartphone or a web server). Firebase^4^ was selected for backend implementation due to its seamless integration with Flutter, providing a real-time database that supports live interactions and collaborative features like push notifications. We additionally employed Google Analytics^5^ to analyze user interaction patterns, facilitating data-driven insights into system acceptance and usage. This technology stack allowed an iterative and incremental development process to integrate feedback from PC practitioners continuously.

By continuously validating and improving our collaboration support system demonstrator, we ultimately derived and refined 13 DRs, organized into five distinct design *areas*, as summarized in Table 3. Following the proposed design principle scheme by Gregor et al. (38), we defined a *mechanism* and *aim* for each DR to ensure we convey our conceptual ideas comprehensively. The mechanism states the action or approach required to achieve the desired aim or how to lead to the expected behavior. The area specifies the broader context in which the corresponding DRs are applied. During our design process, we considered the four constructs: performance expectancy, effort expectancy, social influence, and facilitating conditions presented as direct determinants of user acceptance and usage behavior (7).

**Figure 2:**
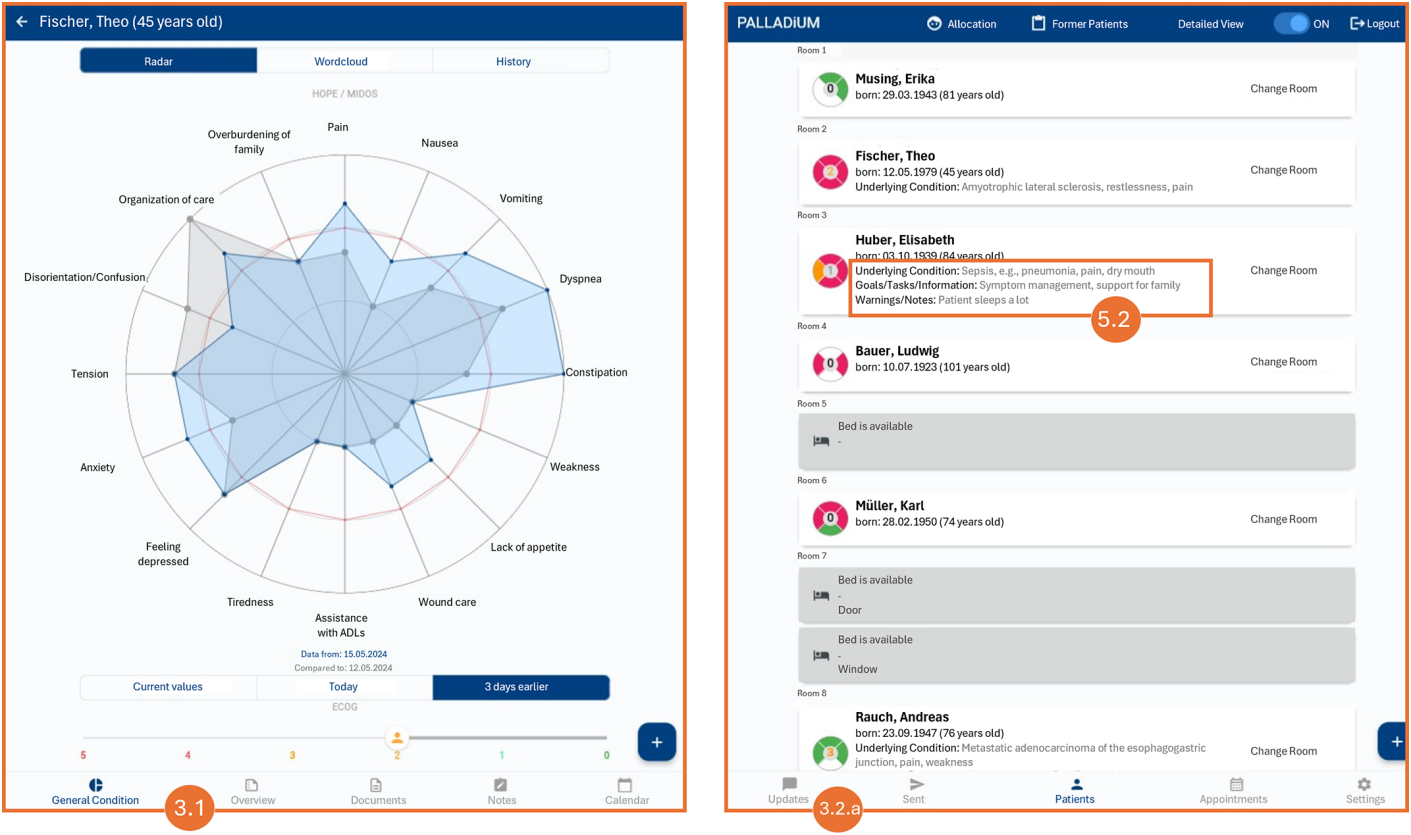
Translated screenshots of the web app. Note: The patient data presented is fictitious.

To ensure a clear understanding of the DRs, we provide a detailed explanation of each area and its DRs and how we integrated them into our demonstrator. The screenshots in Figures 1, 2 illustrate some concrete features implemented using fictional patient data.

#### 3.2.1 Access and usability

As mentioned in Table 1, information and knowledge gaps arise due to deficiencies of the existing HIS and incomplete documentation, resulting in undesired effects such as delays, uncertainty among the PC HCPs, or double documentation. To effectively prevent these effects and promote fast and comprehensive adoption, a multi-professional collaboration support system should facilitate documenting and accessing information and knowledge while ensuring adaptability to the rapidly changing environment. To mitigate the undesired effects of team dynamics or hierarchical structures, it is reasonable to equalize the users regarding the use of information and knowledge. Therefore, we derived the following three DRs to increase access and usability.

To enhance platform accessibility and usability, DR 1.1 recommends seamless collaboration across various portable devices such as phones and tablets. This approach aims to reduce barriers, allowing PC professionals to document their work from any location using their preferred devices. The system's functionalities must adapt to the specific device and operating system to ensure high usability. For our demonstrator, we used the open-source UI software development kit Flutter. It allows building natively compiled mobile, web, and desktop applications from a single codebase. To provide the features in a clear and structured way, we implemented a user-friendly interface that automatically adjusts screen layouts, font sizes, and the amount of information displayed based on the device's display size (see different screen layouts in Figures 1, 2). With this responsive design, our demonstrator can be accessed and used from multiple devices, promoting a user-friendly and intuitive experience across all platforms.

DR 1.2 promotes a unified interface and equal access rights across the system. This aims to democratize the multi-professional collaboration support system by allowing equal and seamless access to information and knowledge to all roles, regardless of profession. Within the context of our demonstrator, each HCP, such as a physician, nurse, psychologist, social worker, or other therapist, has access to the same interface and set of information. With this approach, everyone on the PC team can consistently input data and access the same information and knowledge, preventing misunderstandings between the different professions due to information asymmetry. Displaying the data automatically to all the professions within the PC team ensures all PC HCPs are on an equal footing, streamlining communication and mitigating informational advantage and team friction. This does not necessarily mean that sensitive data is shared with more people than is already the case through communication between PC team members. Instead, it promotes the diversity of documentation and the development of more suitable functions for exchanging information and knowledge for each profession.

By implementing user-friendly search and filter functionality like promoted in DR 1.3, the retrieval of relevant information and knowledge becomes more efficient and user-friendly. In the sense of an enhanced pull rationale, PC professionals are empowered to retrieve the information demanded. In the context of our demonstrator, it is possible to tag documents with relevant keywords that reflect their core content. As a result, the corresponding documents are displayed when searching for concrete information like “first contact” or “shortness of breath.” Another approach is to offer robust search and filtering capabilities based on criteria such as patient, time, keywords, the document's author, and more (Figure 1, Number 1.3). By following this DR, care providers can rapidly locate the exact information they require, reducing the time seeking and mental effort to extract the desired information and knowledge.

#### 3.2.2 Information and knowledge exchange

Information and knowledge asymmetries resulting from different perspectives among PC HCPs or discrepancies between oral communication and written documentation can lead to significant challenges in daily care on a PC ward. The nature of shift work in hospitals enforces these issues since HCPs usually communicate only synchronously, with limited opportunities for asynchronous exchange. This can lead to delays, misunderstandings, or a complete lack of communication. As our empirical findings imply (Table 1), this might negatively affect the caretakers and patients. Enabling PC HCPs to exchange information and knowledge synchronously and asynchronously with additional options to supplement further contexts like urgency, comments, or pending items facilitates information and knowledge flow among PC HCPs. Based on these insights, we have developed the following DRs.

DR 2.1 facilitates the exchange of information and knowledge among HCPs by supporting asynchronous communication. It enables users to send documents like performance recordings, symptom reports, and other forms of medical or therapeutic notes to single professionals, pre-defined groups, or all team members (Figure 1, Number 2.1.a). The received documents and messages are then listed in a news feed so the user can immediately grasp what was recently sent to them (Figure 1, Number 2.1.b). This feature is handy for introducing an interprofessional dialogue, as it allows for the attachment of messages, adding personal highlighting of specific aspects (Figure 1, Number 2.1.c). The attachment is solely visible to the recipient from the pull message to the alert push message, whereas the underlying documentation becomes part of the HIS. This structured and low-threshold approach to information sharing has the dual benefit of enhancing collaboration and continuity of care while ensuring that critical information reaches the relevant individuals efficiently, regardless of, for example, their immediate availability or current focus on other tasks. Additionally, it allows HCPs to weigh their profession-specific setting of significance against those of other team members.

By enabling the real-time sharing of critical information with relevant HCPs, as mentioned in DR 2.2, an immediate and continuous professional exchange is promoted, keeping the team updated on patient conditions and unresolved issues. In our demonstrator, users can indicate the urgency of these messages by choosing between standard and high priority (Figure 1, Number 2.2). Highlighting critical updates with push messages also requests prompt responses or actions. This feature not only enhances the efficiency of information and knowledge dissemination but also supports cohesive and well-coordinated care, as team members involved are kept informed and aligned with the latest and most critical patient information and treatment plans.

An additional approach to enhance collaboration and coordination is to improve transparency regarding the availability and responsibility of professionals and spatial resources (DR 2.3). This can be achieved by clearly outlining care plans and disclosing which professional is responsible for which patient. In the context of our demonstrator, we implemented that approach by enabling the HCPs to assign themselves to the patients for whom they are currently responsible. This information can then be leveraged by others, for example, when sending patient information only to the group of HCPs it concerns. Based on the assignment of the caretaker to the patients, the interface indicates who is currently responsible for the corresponding patient (Figure 1, Number 2.3).

#### 3.2.3 Information and knowledge tangibility

Although healthcare systems already store vast amounts of data, their whole potential is often not fully leveraged. Due to time constraints, HCPs usually do not have the resources to thoroughly review both the extensive historical and current data for each patient or to stay updated on all significant developments in each patient case. As a result, before making informed decisions in multi-professional case discussions, key issues that require attention must be identified, and generally, all participants need to be briefed on the current situation. To address this, we developed DRs that make information and knowledge more tangible, allowing system users to briefly grasp the patient's history and identify the essential facts and developments that require multi-professional discussions.

To help PC HCPs quickly comprehend and interpret complex patient histories, DR 3.1. promotes applying various visual representations such as charts, graphs, and timelines. This aims to leverage past knowledge and support clinicians in quickly grasping the patient's medical journey, identifying patterns and trends, gaining valuable insights rapidly, and making more informed decisions. We incorporated a radar chart to display the multivariate data of the symptoms derived from ‘Hospice and Palliative Care Evaluation—Symptom and Problem Checklist (HOPE-SP-CL)’ (39) and MIDOS (40) in a compressed and synoptic manner (Figure 2, Number 3.1). Within the demonstrator, the current and past observed values can be seen. Moreover, a line chart that can be filtered depending on symptoms illustrates the development of the patient's symptoms over time. Even though the perspective of the patient and their relatives play a vital role in person-centered and consistently needs-focused PC, our focus was not on patient input, as the scope of our study focuses on the collaboration within the PC team; not between patients and PC team members. Symptoms and concerns raised by patients can still be recorded and accessed, for example, in the notes or dialogues of the PC team members (cf. DRs 2.1, 2.2, 4.2, 5.1–5.3), to allow consideration of critical conditions and highlight the need for attention.

As promoted in DR 3.2. processing and aggregating emphasize the careful handling of data to ensure its clarity and reliability. It involves preprocessing unstructured data, such as free-text clinical notes or patient feedback, to transform it into structured formats that can be used for analysis and generate accurate and actionable insights. The system should also highlight discrepancies between various data sources, enabling HCPs to identify and reconcile conflicting information, which is vital for making well-informed decisions. Furthermore, the system should offer synthesized summaries that distill complex information into easily digestible overviews, enabling HCPs to understand critical points at a glance. The patient overview in Figure 2 (Number 3.2.a) displays the current patients, their rooms, and their most important facts. The ‘symptom flower’ in the patients’ overview (Figure 1, Number 3.2.b) immediately shows a patient's current situation by illustrating the current symptom burden classified by the four traditional dimensions of symptoms in PC: physical, psychological, social, and spiritual. The color indicates whether symptoms of a dimension are in critical condition and need attention. Similarly, we utilized a symptom cloud based on structured, routinely collected data (Figure 1, Number 3.2.c). The size and color of the respective symptom indicate the direction and severity of the symptom's development, providing a valuable starting point for further discussions. By applying natural language processing techniques, even more concise and coherent summaries of complex datasets could be generated to enhance information accessibility. However, regarding all processing and aggregation utilization, it is crucial to provide the results in a verifiable manner. This means that users can trace the aggregated data to the original data, enabling them to understand how the outcomes were generated and to validate the results according to accuracy and integrity. Providing transparency in the data aggregation process is particularly critical to strengthen the certainty of interpretation and action, encouraging its adoption and consistent use.

#### 3.2.4 Data integration

The system must always store and display the most up-to-date and correct data to provide a reliable and shared database that supports continuous and accurate information and knowledge exchange while accounting for typical dynamics and rapid situational changes. Achieving this requires that the system integrates data from other sources appropriately, utilizes seamless update processes, and ensures structured digital storage for various types of data like documents, notes, information, and knowledge.

DR 4.1 ensures patient data is centralized, correct, and available, allowing continuous, real-time updates. By implementing multidirectional update processes, the system ensures that data from single-point-of-truth systems like HIS and other systems are accessible, synchronized, and consolidated. This centralization facilitates comprehensive patient data management, ensuring all relevant information is current and easily accessible. Streamlined authentication processes, such as single sign-on, should be applied to enhance security and ease of access. This allows the professionals to efficiently log in and access the necessary data without repeatedly entering credentials.

By allowing users to draw, write, record, store, illustrate, and organize their notes digitally, anywhere and anytime, as promoted in DR 4.2, HCPs are encouraged to build up their personal knowledge base. In contrast to handwritten paper notes, eventually spread over different papers and locations, capturing observations and reflections digitally in one central database enables individuals to organize, keep track, and flexibly return to their notes. While enhancing knowledge retention, knowledge usage is also facilitated and increased.

#### 3.2.5 Documentation flexibility

The multi-professional collaboration support system should also reflect such circumstances since PC is hugely individualized based on the patients and specialized according to the service and team-specific requirements. To address the system's adaptability and flexibility in the documentation processes, we derived the following three DRs.

DR 5.1 promotes system adaptability according to the organizational needs to support palliative-specific documentation. While ensuring the mandatory documentation, our demonstrator allowed users, to some extent, to customize free text fields and adapt the structure of their input to fit the specific context best. Depending on the decision of the respective PC unit, this could involve including social aspects like the patient's perspective on the goals of care or capturing what matters most to the patient. As a result, the individual and multi-professional nature of PC and its processes are maintained and promoted.

To obtain an even more holistic view of patients and their individual needs, DR 5.2 suggests allowing HCPs to capture transaction data that rarely changes. This includes information and knowledge that is neither fixed personal data such as name, date of birth, or origin nor fluid medical data like the RCD. Concrete examples would be information about the family situation, such as a problematic relationship with siblings or that the patient only wants to be treated by female professionals due to her cultural background. Or information about the patient's preferences, dislikes, and individual practices, e.g., the curtain in the room should always be half-closed to avoid too much light. Typically, this information only becomes apparent gradually during treatment and interactions with the patients and their families. However, as this data is often vital throughout the entire duration of PC, it is beneficial to store this data separately in the patient profile (Figure 2, Number 5.2). This approach ensures that other HCPs can access this information and update it if necessary.

Another way to promote flexibility and incorporate individuality is by offering various methods for documenting information and knowledge (DR 5.3). A collaboration support system should include features like a comprehensive recording of decisions via voice recording or multimedia integration of transcriptions, images, or audio. Allowing individuals to choose their preferred style increases their motivation to be more thorough, enhancing overall transparency, accountability, and exchange of information and knowledge. Employing such methods enables fast knowledge codification and thus captures more information and knowledge efficiently since, for example, all decisions and non-decisions can be documented more easily. Another advantage of storing data as audio and text files is that it facilitates automated and meaningful analysis with large language models or natural language processing, as indicated in DR 3.1.

### 3.3 Evaluation

An integral part of our action design research philosophy was the ongoing evaluation process closely aligned with practitioners (formative throughout the project and summative at the end of the project), continuing our co-creation approach. To assess the effectiveness of the demonstrator and the resulting changes and effects in collaboration from the use of the demonstrator (from the team's perspective, observable and objectifiable), we aimed to analyze both the added value and limitations of the functional demonstrator in a practical application context. During a four-day summative evaluation, team members used the app prototype in their daily work routines as practical experts with their respective perspectives and reflections (see method section, stage II). The results of the qualitative data analysis can be summarized into evaluation results *E1–4)*. Each is illustrated with an anchor example from the collected data.

E1) The demonstrator app was used not only for documentation and information transfer but also as a collaboration and communication tool and for knowledge transfer.

Additional message directly to [Team Member]: “*Severe emotional and psychological stress due to disgust, changes in body image, and wound situation!*”

Associated Documentation: “*The patient independently took care of herself in the bathroom this afternoon; she ate after mobilization to the edge of the bed; changed dressing, during which the patient reported mild pain; she expressed strong disgust about the secretions and stated that the changes in body image are causing her significant distress, making her reluctant to leave the room*.” (documentation; field protocol_ evaluation, pos. 159–160; translated by the authors)

E2) In the team, only the everyday practical combination of reading, talking, and personal impression creates a “complete picture.”

The physician points out that it is also challenging to visualize the patients and their symptoms based on the case vignettes alone, and it is essential to see the patients as well. (multidisciplinary team meeting; field protocol_evaluation, pos. 22; translated by the authors).

E3) The overall feedback can vary significantly even within a single professional group.

*“Well, I am a digital native, but still, I took 5 min per card, and I can read on the go. Looking at a note three times a day isn't really…”* (nursing staff; field protocol_evaluation, pos. 114; translated by the authors).

The nurse appears upset and points out that she has not yet addressed the prompts either, saying, *“It's too much for me; it doesn't fit.”* (field protocol_evaluation, Pos. 92; translated by the authors).

E4) Established understandings of ‘good’ work practices present a particular challenge but not an obstacle in the field of PC and must be appropriately considered in technology development.

[NAME] then explains that their work, or the work of therapeutic professional groups, is characterized by a shared attitude (a term mentioned several times and very emphatically) incompatible with technology. Communication often happens personally and between the lines; much is understood without being explicitly stated because people have known each other for so long and well. (psychosocial-therapeutic professional group, field protocol_evaluation, Pos. 23; translated by the authors).

While the research team is sitting together in the living room discussing the first day of evaluation, [NAME] joins in and says, *“I think the idea of using the smartphone is great; I can very well imagine how it will all work once it's fully operational.”* [NAME] positively highlights that one can *“document everywhere”* and *“track”* what is happening. (psychosocial-therapeutic professional group; field protocol_evaluation, Pos. 33; translated by the authors).

The evaluation of the demonstrator app provides valuable insights into its potential and areas for further focus. The results reveal promising trends towards a comprehensive use of the app, which can inform both its ongoing development and future research. Key findings indicate that integrating the app into daily practice significantly impacts documentation, information and knowledge transfer, as well as team interaction. The variability in feedback across professional groups also emphasizes the necessity for considerable flexibility to ensure broad team acceptance.

## 4 Discussion

### 4.1 Contribution

One of the biggest challenges in the field of PC is the integration of diverse professional perspectives into a unified care approach that prioritizes the patient and their next of kin. The diversity of HCPs, with varying educational backgrounds, specializations, hierarchical levels, and areas of expertise, gives rise to many interpretations regarding the same clinical situation. Each professional domain, such as nursing, psychology, spiritual care, physical therapy, or medicine, contributes its distinctive knowledge and skills when assessing clinical observations. This diversity is beneficial in creating a more holistic understanding of the patient's needs; however, it also challenges reconciling differing viewpoints. In the context of digitalization, these challenges present opportunities for research and development aimed at designing tools that support the synthesis of these diverse perspectives into actionable insights. We recommend that future digital systems (e.g., HIS) facilitate enhanced integration and representation of professional knowledge in a comprehensive and accessible manner. This necessity is particularly crucial in the context of PC, where interdisciplinary collaboration is indispensable for providing comprehensive and holistic care. Therefore, this study presents recommendations for designing digitally supported multi-professional collaboration for the first time. Our comprehensive stakeholder-oriented action design research approach strengthens the results. Integrating knowledge (as distinct from mere information) and a consistently co-creational approach opens up novel technical development processes that more precisely address the specific field of action and can enhance user acceptance. These processes lead to the creation of new, field-specific technical artifacts, which, in turn, pave the way for new information, knowledge, and interaction pathways. However, until now, empirically based recommendations on designing digital systems in this context were missing. The results can also inform research and development in other healthcare fields, such as intensive care, where similar challenges of multi-professional collaboration might be addressed with knowledge-driven digitalization.

In the context of an interprofessional discourse, the presence of traditional hierarchical structures in healthcare can introduce a bias whereby observations and interpretations from different professional backgrounds are weighed disproportionately in a joint discussion. Such a situation may result in misinterpretation and the formulation of therapeutic decisions that are more aligned with the aforementioned hierarchy and less aligned with the patient's needs, ultimately leading to less effective collaboration of the PC team in the long term. Digitalization of healthcare following our proposed recommendations has the potential to solve this issue by enabling the presentation of observations and considerations from various professional groups in an egalitarian manner, independent of traditional healthcare hierarchies. This encourages interprofessional dialogue and facilitates reflection on the existing hierarchical structures within and beyond the patient, who is the subject of the discussion.

### 4.2 Outlook

The limitations of this approach are evident both within and beyond the context of PC. The scalability of findings in PC regarding promoting an egalitarian approach to collaboration may be constrained by the fact that the background in other areas of healthcare differs from that in PC. In PC, open and egalitarian discussion is a valued aspect of collaboration, although this is not always followed up. Furthermore, other areas of healthcare may, in contrast, benefit in certain circumstances from hierarchical structures, like facilitating timely decision-making and/or clarifying responsibilities. Therefore, the applicability and scalability of digital interventions designed to promote egalitarian discourse in teams must always be considered in the context of the specific collaboration scenario. Nevertheless, the study's strength lies in its ability to sensitize to this opportunity and challenge, as well as in the rigorous societal research conducted in conjunction with the research and development of the collaboration support system demonstrator. Formative evaluation of the process and products of the research and development process, with awareness of the influence on established hierarchical structures in healthcare, can facilitate acceptance and integration. Furthermore, it can promote dialogue beyond the digitalization initiative about the means and attitudes of collaboration. Here, more research is required to extend our understanding of the impact of digitalization on hierarchical structures in healthcare and to consider the potential for digitalization strategies to support established hierarchical structures.

In clinical practice, documentation within health records bridges the temporal and spatial gaps that arise from asynchronous communication, effectively replacing the need for direct, personal conversations about specific patient-related topics. However, a substantial proportion of communication is non-verbal, which is lost when transitioning to a purely written format, as is the case with clinical health record documentation. Furthermore, clinical health record documentation serves several functions beyond merely providing information to colleagues. These include the fulfillment of legal and billing requirements, which can dilute the focus on clinical communication. The provision of opportunities for HCPs to emphasize critical information for relevant team members and to provide explanations or comments on the documentation considering the recipient's tasks may facilitate the transfer of information and enhance the clarity of the communicator's perspective. Nevertheless, even with these improvements, textual communication remains a more time-consuming process for the writer and the reader. Further research is required to investigate how incorporating the writer's background, including previously generated documentation from the healthcare team and the writer's personal history, could enhance the clarity and relevance of information. Nevertheless, this approach has limitations. It may encourage specific HCPs to overly contextualize and personalize their notes, which could lead to difficulties for recipients in prioritizing tasks and maintaining workflow efficiency. Furthermore, constant exposure to personalized information and requests could overwhelm recipients and disrupt their task management. This is particularly relevant when patients enter further information into the system, such as self-reported symptom burden and personal goal setting. Therefore, future research should also investigate connecting patients and/or families by integrating their inputs to increase knowledge gain for the multi-professional team.

Digital systems should incorporate features that facilitate the real-time aggregation and contextualization of multi-professional inputs, for instance, by leveraging natural language processing. In PC, where emotional, physical, spiritual, and psychological aspects converge, the availability of tools that facilitate the integration of these perspectives without introducing bias will be of significant benefit. These principles also have the potential to be applied beyond the healthcare field, providing a model for developing collaboration support systems in other domains where the harmonization of diverse expertise is required, such as social services or multidisciplinary project management. In conclusion, the recommendations formulated as a result of our study represent an initial step towards establishing a framework for the research and development of knowledge-driven digitalization in PC. Its scalability varies from topic to topic, but for many other areas of healthcare specialization, PC may provide a promising template for identifying and addressing ubiquitous challenges.

## Data Availability

The analyzed data cannot be made available because, despite pseudonymization, it allows conclusions to be drawn about the persons and their functions within the institution under observation. Requests to access these datasets should be directed to Werner Schneider, werner.schneider@phil.uni-augsburg.de.

## Footnotes

^1^As part of the PALLADiUM research project, a German-language video about the demonstrator was produced. It can be accessed via the following link: https://youtu.be/y0gr3dTgcbo.

^2^ https://flutter.dev/

^3^ https://dart.dev/

^4^ https://firebase.google.com/

^5^ https://developers.google.com/analytics

## References

[1] Deutsche Krebsgesellschaft, Deutsche Krebshilfe, Arbeitsgemeinschaft der Wissenschaftlichen Medizinischen Fachgesellschaften. Erweiterte S3-Leitlinie Palliativmedizin für Patienten mit Einer Nicht-heilbaren Krebserkrankung. Berlin: Deutsche Gesellschaft für Palliativmedizin e. V. (2020). Available at: https://www.dgpalliativmedizin.de/images/stories/pdf/LL_Palliativmedizin_Langversion_2.2.pdf (Accessed February 13, 2025).

[2] Payne S Harding A Williams T Ling J Ostgathe C. Revised recommendations on standards and norms for palliative care in Europe from the European association for palliative care (EAPC): a delphi study. Palliat Med. (2022) 36(4):680–97. 10.1177/02692163221074547 35114839 PMC9006395

[3] Radbruch L SAP. White paper on standards and norms for hospice and palliative care in Europe: part 1. Eur J Palliat Care. (2010) 17:22–33.

[4] Wöhl M Gimpel H Meindl O Ostgathe C Peuten S Schneider W Boosting multi-professional collaboration in palliative care through digital technologies: an action design research study. Bus Inf Syst Eng. (2024). 10.1007/s12599-024-00897-0

[5] Rouidi M Elouadi AE Hamdoune A Choujtani K Chati A. TAM-UTAUT and the acceptance of remote healthcare technologies by healthcare professionals: a systematic review. Inform Med Unlocked. (2022) 32:101008. 10.1016/j.imu.2022.101008

[6] Davis FD. Perceived usefulness, perceived ease of use, and user acceptance of information technology. MIS Q. (1989) 13(3):319. 10.2307/249008

[7] Venkatesh V Morris M Davis G. User acceptance of information technology: toward a unified view. MIS Q. (2003) 27(3):425. 10.2307/30036540

[8] Schröder J Riiser K Holmen H. Healthcare personnel’s perspectives on health technology in home-based pediatric palliative care: a qualitative study. BMC Palliat Care. (2024) 23(1):137. 10.1186/s12904-024-01464-w 38811957 PMC11134737

[9] Keenan J Rahman R Hudson J. Exploring the acceptance of telehealth within palliative care: a self-determination theory perspective. Health Technol (Berl). (2021) 11(3):575–84. 10.1007/s12553-021-00535-9

[10] Ndayizigamiye P Maharaj M. Mobile health adoption in Burundi: a UTAUT perspective. 2016 IEEE Global Humanitarian Technology Conference (GHTC) IEEE. (2016). p. 613–23

[11] Burner-Fritsch I Busse TS Deckers M Giehl C Kulla A Muente C Digitalisierung in der Palliativversorgung: Chancen und Herausforderungen. Berlin: Deutsche Gesellschaft für Palliativmedizin e.V. (2022). Available at: https://www.dgpalliativmedizin.de/images/221121_Arbeitspapier_Digitalisierung.pdf (Accessed February 13, 2025).

[12] Ott T Heckel M Öhl N Steigleder T Albrecht NC Ostgathe C Palliative care and new technologies: the use of smart sensor technologies and its impact on the total care principle. BMC Palliat Care. (2023) 22(1):50. 10.1186/s12904-023-01174-9 37101258 PMC10131446

[13] Suslow A Giehl C Hergesell J Vollmar HC Otte I. Impact of information and communication software on multiprofessional team collaboration in outpatient palliative care: a qualitative study on providers’ perspectives. BMC Palliat Care. (2023) 22(1):19. 10.1186/s12904-023-01141-4 36882733 PMC9991877

[14] May S Bruch D Gehlhaar A Linderkamp F Stahlhut K Heinze M Digital technologies in routine palliative care delivery: an exploratory qualitative study with health care professionals in Germany. BMC Health Serv Res. (2022) 22(1):1516. 10.1186/s12913-022-08802-9 36514156 PMC9745710

[15] Thiebes S Gao F Briggs RO Schmidt-Kraepelin M Sunyaev A. Design concerns for multiorganizational, multistakeholder collaboration: a study in the healthcare industry. J Manag Inform Syst. (2023) 40(1):239–70. 10.1080/07421222.2023.2172771

[16] Ali D A Meniari A E Filali S E Morabite O Senhaji F Khabbache H. Empirical research on technological pedagogical content knowledge (TPACK) framework in health professions education: a literature review. Med Sci Educ. (2023) 33(3):791–803. 10.1007/s40670-023-01786-z 37501808 PMC10368588

[17] Depper T. Organisation und Wissen. In: Schützeichel R, editor. Handbuch Wissenssoziologie und Wissensforschung. Köln: Herbert von Halem Verla (2007). p. 588–612.

[18] Tsoukas H Mylonopoulos N. Organizations as Knowledge Systems. London: Palgrave Macmillan (2004).

[19] Porschen S. Austausch Impliziten Erfahrungswissens. Wiesbaden: VS Verlag für Sozialwissenschaften (2008).

[20] Schneider W Stadelbacher S. Palliative Care und Hospiz. In: Kriwy P Jungbauer-Gans M, editors. Handbuch Gesundheitssoziologie. Wiesbaden: Springer VS (2020). p. 481–509.

[21] Vargas C Whelan J Brimblecombe J Allender S. Co-creation, co-design, co-production for public health: a perspective on definition and distinctions. Public Health Res Pract. (2022) 32(2):e3222211. 10.17061/phrp3222211 35702744

[22] Giannitrapani KF Lin K Hafi LA Maheta B Isenberg SR. Codesign use in palliative care intervention development: a systematic review. J Pain Symptom Manage. (2024) 68(4):e235–53. 10.1016/j.jpainsymman.2024.06.007 38909694

[23] Wright MT Brito I Cook T Harris J Kleba ME Madsen W What is Participatory Health Research?: Position Paper No. 1. Berlin: International Collaboration for Participatory Health Research (ICPHR). (2013).

[24] Ertz M. Co-creation. Encyclopedia. (2024) 4(1):137–47. 10.3390/encyclopedia4010012

[25] Monnard K Benjamins MR Hirschtick JL Castro M Roesch PT. Co-Creation of knowledge: a community-based approach to multilevel dissemination of health information. Health Promot Pract. (2021) 22(2):215–23. 10.1177/1524839919865228 31470741

[26] Grindell C Coates E Croot L O'Cathain A. The use of co-production, co-design and co-creation to mobilise knowledge in the management of health conditions: a systematic review. BMC Health Serv Res. (2022) 22(1):877. 10.1186/s12913-022-08079-y 35799251 PMC9264579

[27] Li M Tuunanen T. Information technology–supported value co-creation and co-destruction via social interaction and resource integration in service systems. J Strategic Inform Syst. (2022) 31(2):101719. 10.1016/j.jsis.2022.101719

[28] Grimminger S Heckel M Markgraf M Peuten S Wöhl M Gimpel H Palliative care as a digital working world (PALLADiUM): a mixed-method research protocol. BMC Palliat Care. (2023) 22(1):102. 10.1186/s12904-023-01173-w 37481524 PMC10362664

[29] Sein M Henfridsson O Purao S Rossi M Lindgren R. Action design research. MIS Q. (2011) 35(1):37. 10.2307/23043488

[30] Black GB van Os S Machen S Fulop NJ. Ethnographic research as an evolving method for supporting healthcare improvement skills: a scoping review. BMC Med Res Methodol. (2021) 21(1):274. 10.1186/s12874-021-01466-9 34865630 PMC8647364

[31] Cubellis L Schmid C von Peter S. Ethnography in health services research: oscillation between theory and practice. Qual Health Res. (2021) 31(11):2029–40. 10.1177/10497323211022312 34286610 PMC8552374

[32] Hammersley M Atkinson P. Ethnography. London: Taylor & Francis Group. (2019).

[33] Strauss A. Grounded Theory in Practice. Thousand Oaks, CA: SAGE Publications, Inc. (1997).

[34] Corbin J Strauss A. Basics of Qualitative Research: Techniques and Procedures for Developing Grounded Theory. Thousand Oaks, CA: SAGE Publications, Inc. (2008).

[35] Bryant A Charmaz K. The SAGE Handbook of Grounded Theory. London: SAGE Publications Ltd (2007).

[36] Corbin JM Strauss A. Grounded theory research: procedures, canons, and evaluative criteria. Qual Sociol. (1990) 13(1):3–21. 10.1007/BF00988593

[37] Phillips L Frølunde L Christensen-Strynø MB. Confronting the complexities of “co-production” in participatory health research: a critical, reflexive approach to power dynamics in a collaborative project on Parkinson’s dance. Qual Health Res. (2021) 31(7):1290–305. 10.1177/10497323211003863 33899575

[38] Gregor S Kruse L Seidel S. Research perspectives: the anatomy of a design principle. J Assoc Inform Syst. (2020) 21:1622–52. 10.17705/1jais.00649

[39] Stiel S Pollok A Elsner F Lindena G Ostgathe C Nauck F Validation of the symptom and problem checklist of the German hospice and palliative care evaluation (HOPE). J Pain Symptom Manage. (2012) 43(3):593–605. 10.1016/j.jpainsymman.2011.04.021 22071164

[40] Stiel S Matthes ME Bertram L Ostgathe C Elsner F Radbruch L. Validation of the new version of the Minimal Documentation System (MIDOS) for patients in palliative care: the German version of the Edmonton Symptom Assessment Scale (ESAS). Schmerz. (2010) 24(6):596–604. 10.1007/s00482-010-0972-5 20882300

